# Supplementary figures and images for: Integrative analysis of long non-coding RNA and mRNA in broilers with valgus-varus deformity
Source: PLoS One. 2020 Sep 24;15(9):e0239450. doi: 10.1371/journal.pone.0239450 (PMC7514040; doi:10.1371/journal.pone.0239450)

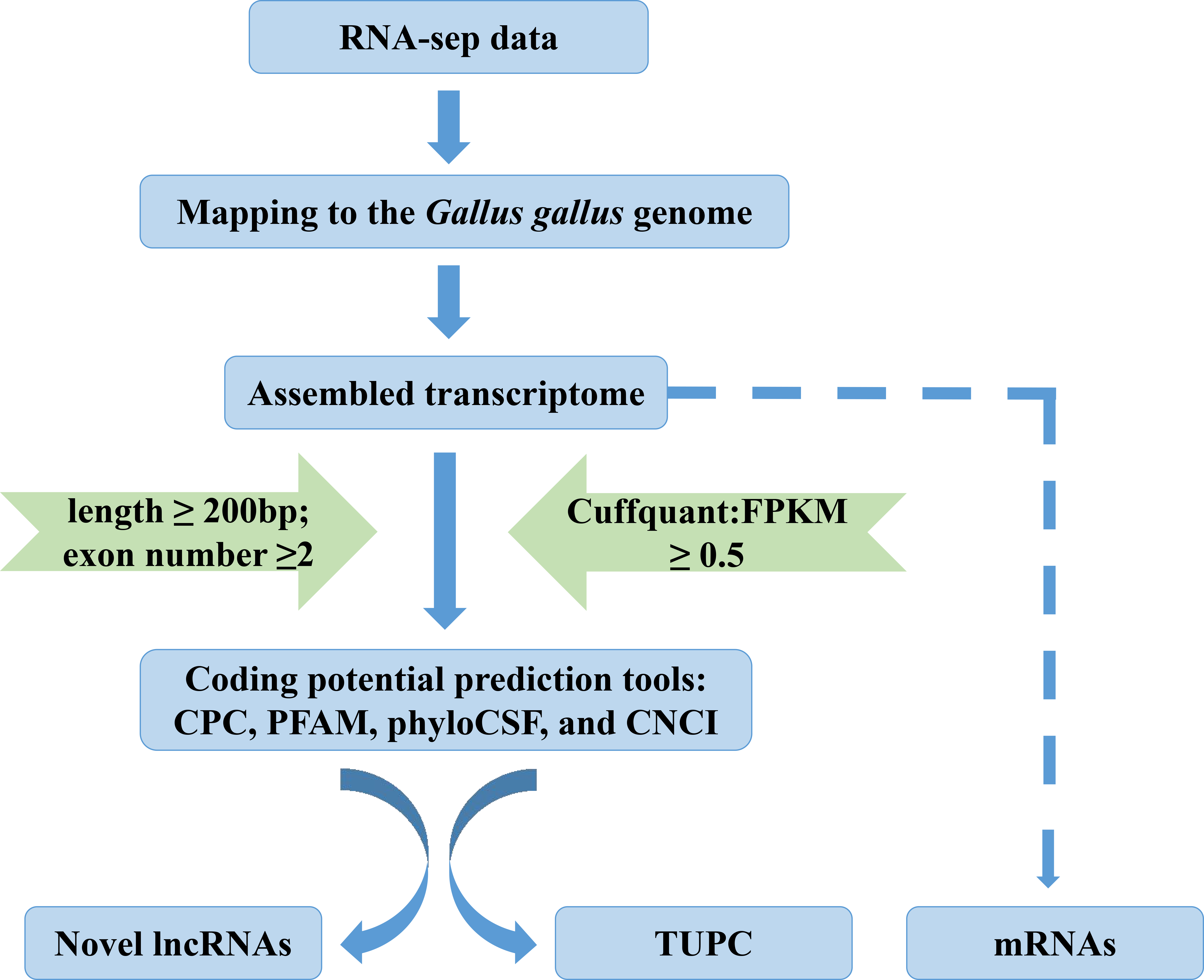

Supplement: S1 Fig — (TIF) [file pone.0239450.s001.tif]

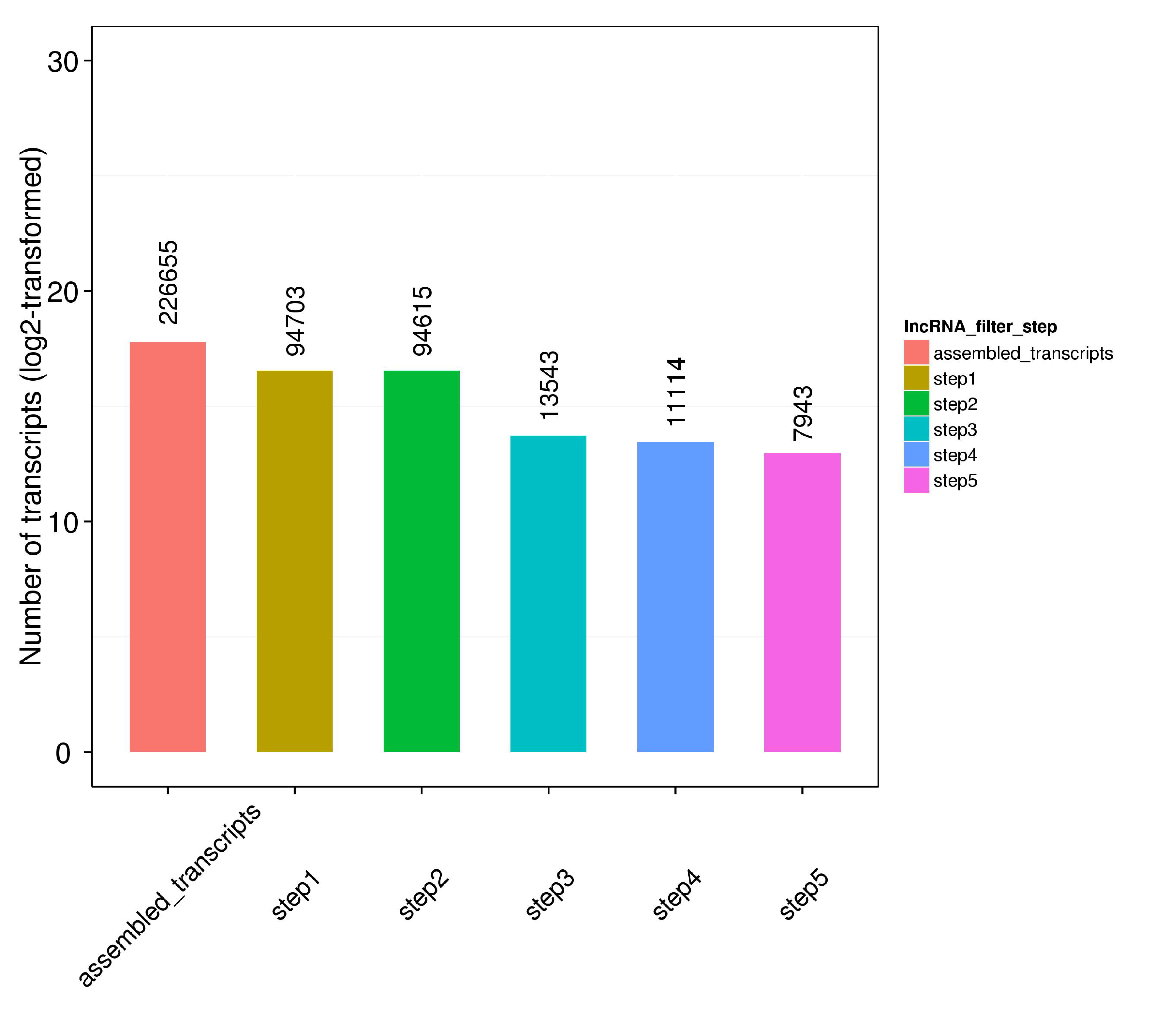

Supplement: S2 Fig — (TIF) [file pone.0239450.s002.tif]
