# Supplementary material for: Integrative analysis of long non-coding RNA and mRNA in broilers with valgus-varus deformity
Source: PLoS One. 2020 Sep 24;15(9):e0239450. doi: 10.1371/journal.pone.0239450 (PMC7514040; doi:10.1371/journal.pone.0239450)
Supplement: Attachment — Submitted filename: Response to Reviewers.docx [file pone.0239450.s011.docx]

Response to Review Comments

Dear editor and reviewer,

Thank you for your letter and the reviewers’ comments concerning our manuscript entitled “Integrative Analysis of Long Non-coding RNA and mRNA in Broilers with Valgus-varus Deformity” (PONE-D-20-04909). We have revised on our manuscript according to reviewer(s)' comments. These comments were valuable and helpful for revising and improving our paper as well as for providing important overall guidance for our study. We have studied the comments carefully and have made corrections, which we hope will meet with your approval. In order to facilitate your examination, any changes we make to the original manuscript is highlighted in red in our revised manuscript.

**The response to comments from Reviewer 1**

**Point 1:** Methods - Were the birds stunned prior to jugular vein severance?

**Response 1:** Thank you for your comments. We apologize for not having a clear description in ethics statement. In fact, the animals were anesthetized with pentobarbitone sodium before the jugular vein severance. We have rephrase the sentence to make it more accurate and clear to understand. The contents are as follows：

**Ethics statement**

The experiments were performed according to guidelines and protocols approved by the Institutional Animal Care and Use Committee of Henan Agricultural University, and were approved by the Animal Care Committee of the College of Animal Science and Veterinary Medicine, Henan Agricultural University, China (18-0120). In order to complying with animal welfare guidelines and minimize animal suffering, animals were euthanized with pentobarbitone sodium before tissue sampling. **(Page 5, Lines 83-86 and line 91)**

**Point 2:** Were any power equations undertaken to assess if 3 birds per group would give representative and accurate data?

**Response 2:** Thank you so much for your comments. We chose 3 birds per group based on this research purpose and budget reasons. In addition, according to the references (*Li et al., 2019; Wang et al., 2019; Wang et al., 2019; Guo et al., 2020*), three biological replicates are feasible for RNA-seq. And after sequencing, we also examined the correlation of mRNA expression levels between different samples. As shown in the figure below, the results show that there is a high correlation coefficient between the three individuals. This suggests that this approach can also achieve our research objective.

References:

*Li Y, Chen Y, Jin W, et al. Analyses of MicroRNA and mRNA Expression Profiles Reveal the Crucial Interaction Networks and Pathways for Regulation of Chicken Breast Muscle Development [J]. Frontiers in genetics, 2019.*

*Wang X, Li D, Song S, et al. Combined transcriptomics and proteomics forecast analysis for potential genes regulating the Columbian plumage color in chickens [J]. PLos One. 2019, 14(11): e0210850.*

*Wang C, Ma W. Hypothalamic and pituitary transcriptome profiling using RNA-sequencing in high-yielding and low-yielding laying hens [J]. Science Reports. 2019, 9(1):10285.*

*Guo Y; Su A; Tian H, et al. Transcriptomic Analysis of Spleen Revealed Mechanism of Dexamethasone-Induced Immune Suppression in Chicks [J]. Genes. 2020, 11(5), 513*

**The response to comments from Reviewer 2**

**Point 1:** This paper covers an area of global interest, due to the large number of birds intensively reared globally for meat and the lameness issues associated with this type of meat production. Please include information on the number of birds produced annually, the strains of birds must commonly use, and the incidence of specific types of lameness in broilers.

**Response 1:** Thank you for your comments. According to your suggesting, we have added the number of birds produced annually, the strains of birds must commonly use and the incidence of specific types of lameness in broilers. The contents are as follows：

The global broiler production was reported to be about 100 million tons in 2019, and the main broiler breeds include Arbor Acres broilers, Avian broilers, Ross 308 broilers, Roman broilers, Cobb broilers, Hubbard broilers. Because of its large size, fast growth rate, high uniformity, and high feed conversion characteristics occupy the main market. However, with the development of the breeding industry towards modernization and intensification, the production efficiency of commercial broilers has been greatly improved, and the broiler leg disease has become increasingly prominent (*Bradshaw RH et al., 2002*). **(Page 3, Lines 2-9)**

Julian (1984) showed that the incidence of VVD in broilers was 0.5-2%. Cruickshank (1986) showed that the incidence of VVD in broilers was 0.4% at 1 week of age and increased to 5.4% at 5 weeks of age. In our previous study of VVD in Hubbard broilers, the incidence was 1.75% *( Julian R et al., 1984; Cruickshank et al., 1986; Guo et al., 2019*). **(Page 3, Lines 19-23)**

References:

*Bradshaw RH, Kirkden RD, Broom DM. A Review of the Aetiology and Pathology of Leg Weakness in Broilers in Relation to Welfare. Avian & Poultry Biology Reviews. 2002. 13 (2): 45-103*

*Julian R J. Valgus-Varus Deformity of the Intertarsal Joint in Broiler Chickens[J]. Canadian Veterinary Journal, 1984, 25(6):254-258.*

*Cruickshank J J, Sim J S. Morphometric and radiographic characteristics of tibial bone of broiler chickens with twisted leg disorders [J]. Avian Diseases, 1986, 30(4):699-708.*

*Guo Y P, Tang H H, Wang X N, et al. Clinical assessment of growth performance, bone morphometry, bone quality, and serum indicators in broilers affected by valgus-varus deformity. Poultry Science. 2019;98(10).*

**Point 2:** Reference to a country (UK) is not appropriate in a paper that is posing a hypothesis that genetic strain predisposes to VVD: the UK does not have unique broiler genetics, it draws from the same key global genetic resources as other countries.

**Response 2:** Thanks for the comments. We have revised them according to your notes. The contents are as follows：

The heritable nature and bone quality traits of VVD have been reported. Heritability of leg problems ranged from 0.10 to 0.13 and that of bone quality ranged from 0.10 to 0.77 (*Bihan et al., 1997; Kapell et al., 2012; Rekaya et al., 2013; Gonzalez et al., 2015*). And the prevalence of VVD has declined through genetic selection. **(Page 12, Lines 338-341)**

References:

*Bihan-Duval E L, Beaumont C, Colleau J J. Estimation of the genetic correlations between twisted legs and growth or conformation traits in broiler chickens[J]. journal of animal breeding & genetics, 1997, 114(1-6):239-259.*

*Kapell D N R G, Hill W G, Neeteson A M, et al. Twenty-five years of selection for improved leg health in purebred broiler lines and underlying genetic parameters[J]. Poultry science, 2012, 91(12):3032-3043.*

*Rekaya R, Sapp R L, Wing T, et al. Genetic evaluation for growth, body composition, feed efficiency, and leg soundness. [J]. Poultry science, 2013, 92(4):923-929.*

*Gonzalez-Ceron F, Rekaya R, Anthony N B, et al. Genetic analysis of leg problems and growth in a random mating broiler population[J]. Poultry science, 2015, 94(2):162-168.*

**Point 3:** Paper focuses on just one strain of broiler and draws conclusions about broilers generally, the paper needs to ensure that the introduction and conclusions make it clear that just one strain of broiler has been investigated.

**Response 3:** We apologize for not describing this clearly. The experimental broiler we selected was Hubbard commercial broiler. And according to your comments, a clearly description has revised in the introduction and conclusions. The contents are as follows：

The Hubbard broiler has a high rate of growth and meat production, and the incidence of leg disease is relatively high. In this study, the transcriptome profiles of spleen tissue from 3 normal Hubbard commercial broilers and 3 VVD Hubbard commercial broilers were compared. **(Page 4, Lines 53-56)**

This study used RNA-Seq technology to analyze the spleen transcriptome in VVD Hubbard broilers and normal Hubbard broilers. The results revealed lncRNA-mRNA networks, novel genes and pathway enrichment that involved in immunity and bone development about Hubbard broilers. These findings will provide some reference for the etiology and pathogenesis of VVD. **(Page 13, Lines 408-410)**

**Point 4:** Introduction does not adequately introduce VVD to the reader.

**Response 4:** Thank you for your comments. According to your comments, we added the description in the introduction, the contents are as follows：

Valgus-varus angulation was classified as normal, mild deformity, intermediate deformity and severe deformity, (tibia-metatarsus angle deviation less than 5° was normal, score = 0; angle between 10° and 25° was mild deformity, score = 1; angle between 25° and 45° was moderate deformity, score = 2; angle greater than 45° was severe deformity, score = 3) (*Shim* et al., 2012; *Gonzalez* et al., 2015). **(Page 3, Lines 11-15)**

Julian (1984) showed that the incidence of VVD in broilers was 0.5-2%. Cruickshank (1986) showed that the incidence of VVD in broilers was 0.4% at 1 week of age and increased to 5.4% at 5 weeks of age. In our previous study of VVD in Hubbard broilers, the incidence was 1.75% *( Julian et al., 1984; Cruickshank et al., 1986; Guo et al., 2019*). Reduction of the incidence and severity of VVD is an urgent matter. Leterrier and Nys have explored the clinical and anatomical differences of broilers with VVD ( *Leterrier et al., 1992*). We also studied the phenotypes and pathological changes in the early stage of development ( *Guo et al., 2019*) . The occurrence of VVD may be influenced by many factors, such as the lighting schedule, overfeeding, lack of exercise and genetics ( *Leterrier et al., 1996*). The specific etiology and pathogenesis of VVD has not been clarified. However, previous studies have shown that VVD carries a certain genetic potential, and the incidence of VVD could be reduced by genetic selection (*Bihan et al., 1996*) **(Page 3, Lines 19-30)**

References:

*Shim MY, Karnuah AB, Anthony NB, Pesti GM, Aggrey SE. The effects of broiler chicken growth rate on valgus, varus, and tibial dyschondroplasia. Poultry Science. 2012;91(1):62-5.*

*Gonzalez-Ceron F, Rekaya R, Anthony NB, Aggrey SE. Genetic analysis of leg problems and growth in a random mating broiler population. Poultry Science. 2015;94(2):162-8.*

*Julian R J. Valgus-Varus Deformity of the Intertarsal Joint in Broiler Chickens[J]. Canadian Veterinary Journal, 1984, 25(6):254-258.*

*Cruickshank J J, Sim J S. Morphometric and radiographic characteristics of tibial bone of broiler chickens with twisted leg disorders [J]. Avian Diseases, 1986, 30(4):699-708.*

*Guo Y P, Tang H H, Wang X N, et al. Clinical assessment of growth performance, bone morphometry, bone quality, and serum indicators in broilers affected by valgus-varus deformity. Poultry Science. 2019;98(10).*

**Point 5:** Bird rearing conditions require the same level of detail as the molecular methodologies, the methodology does not adequately describe the rearing conditions of the birds used.

**Response 5:** Thanks for the comments. According to your comments, we added the bird rearing conditions description as follows:

**Anima****ls and Management**

Hubbard broilers (n = 52,000) were reared at a commercial farm in Tangshan for 40 days. Cage dimensions were 0.8 m long, 0.8 m wide and 0.35 m high. Stocking density was 30 birds when 1 to 9 days old and 12 birds aging 10 to 40 days old. The temperature of the newly hatched chicken house should be 29℃-33℃, then it should be reduced 2℃-3℃ per week, and to 21 ℃ at the age of 4 weeks, and the temperature in the late fattening period should not be lower than 18℃. In the first two weeks, the relative humidity of the chicken house should be controlled at 65-70%, and in the later period it should be controlled at 55-60%. Lighting was maintained at 60 lux 24L (0 to 5 days), 8 lux 17L (6 to 32 days), and 60 lux 24 L (33 to 40 days). All broilers were fed uniform diets containing 1.0% Ca, 0.45% P, 2,950 kcal/kg ME, and 21.5% CP at age 1 to 18 days; 0.85% Ca, 0.38% P, 3,100 kcal/kg ME, and 20.0% CP at age 19 to 40 days. Broilers were injected with newcastle disease vaccine, mycoplasma vaccine and infectious bursal disease virus vaccine at 1 day old; And immunized with nd-ib vaccine by drinking water at 7 days old and the newcastle disease vaccine was injected at 21 days of age. **(Page 4, Lines 64-79)**

**Point 6:** Euthanasia method does not comply with the PLOS1 requirements for adherence to AVMA guidelines for euthanasia of animals

**Response 6:** Thank you for your comments. We apologize for not having a clear description in ethics statement. In fact, the animals were anesthetized with pentobarbitone sodium before the jugular vein severance. We have rephrase the sentence to make it more accurate and clear to understand. The contents are as follows:

**Ethics statement**

The experiments were performed according to guidelines and protocols approved by the Institutional Animal Care and Use Committee of Henan Agricultural University, and were approved by the Animal Care Committee of the College of Animal Science and Veterinary Medicine, Henan Agricultural University, China (18-0120). In order to complying with animal welfare guidelines and minimize animal suffering, animals were euthanized with pentobarbitone sodium before tissue sampling. **(Page 5, Lines 83-86 and line 91)**

**The response to comments from Reviewer 3**

**Point 1:** The manuscript describes a useful insight into molecular mechanism and parthenogenesis of VVD. This study can add to genetic selection of broilers to reduce its prevalence. However, the number of samples is insufficient and more samples needed

**Response 1:** Thank you for your valuable comments. We chose 3 birds per group based on this research purpose and budget reasons. In addition, according to the references *(Li et al., 2019; Wang et al., 2019; Wang et al., 2019; Guo et al., 2020)*, three biological replicates are feasible for RNA-seq. And after sequencing, we also examined the correlation of mRNA expression levels between different samples. As shown in the figure below, the results show that there is a high correlation coefficient between the three individuals. This suggests that this approach can also achieve our research objective. In addition, we chose 3 birds per group based on this research purpose and budget reasons.

References:

*Li Y, Chen Y, Jin W, et al. Analyses of MicroRNA and mRNA Expression Profiles Reveal the Crucial Interaction Networks and Pathways for Regulation of Chicken Breast Muscle Development [J]. Frontiers in genetics, 2019.*

*Wang X, Li D, Song S, et al. Combined transcriptomics and proteomics forecast analysis for potential genes regulating the Columbian plumage color in chickens [J]. PLos One. 2019, 14(11): e0210850.*

*Wang C, Ma W. Hypothalamic and pituitary transcriptome profiling using RNA-sequencing in high-yielding and low-yielding laying hens [J]. Science Reports. 2019, 9(1):10285.*

*Guo Y; Su A; Tian H, et al. Transcriptomic Analysis of Spleen Revealed Mechanism of Dexamethasone-Induced Immune Suppression in Chicks [J]. Genes. 2020, 11(5), 513*

**The response to comments from Reviewer 4**

**Point 1:** The authors should add the relation between VVD and spleen, and declare the significance of spleen transcript sequencing for VVD.

**Response 1:** Thank you for your valuable comments. According to your comments, we have made a further supplement to the paper and the contents are as follows:

The description " A number of studies have analyzed the effects of disease on spleen transcriptomics through high-throughput sequencing. As the hub of the neural-endocrine-immune network, the spleen contains a variety of immune active cells and immune factors. Abnormal immune system function will cause bone metabolism disorder, and then change bone homeostasis, leading to the occurrence of disease. T cells are one of the most important immune cells in the immune response of the spleen, in addition to playing a role in the immune system, it also participates in the process of bone reconstruction, which has a certain influence on the formation of osteoclasts (*Takayanagi, 2005; Arron et al.,2000*). And previous studies have found that abnormal changes of bone histomorphology in VVD broilers." has been added in the revised manuscript. **(Page 4, Lines 44-53)**

References:

*Takayanagi H. Inflammatory bone destruction and osteoimmunology. Journal of Periodontal Research. 2005;40(4):287-93.*

*Arron JR, Choi Y. Bone versus immune system. Nature. 2000;408(6812):535-536.*

**Point 2:** Does the sound broilers mean the normal broilers? There were two statements in the paper, you should keep it consistent.

**Response 2:** In the paper, the sound broilers mean the normal broilers, we apologize for not keep it consistent, and it has been revised one by one in the paper. Thank you.

**Point 3:** Since, it is an RNA seq, the authors must provide submission links where the RNAseq raw data is submitted.

**Response 3:** Thank you for your valuable comments. We have submitted the data to NCBI SRA. The RNA-Seq datasets supporting the conclusions of this article are available at NCBI project PRJNA644292 with accession number SRR12153415, SRR12153416, SRR12153417, SRR12153418, SRR12153419, SRR12153420. **(Page 5, Lines 113-116)**

**Point 4:** The format of references need modification, for example, L406, L416, L497……

**Response 4:** Thank you very much for your careful review. According to your comments, we have reviewed the whole references and modified the format of references that don't meet the requirements.

**Journal Requirements:**

When submitting your revision, we need you to address these additional requirements.

We have improved the manuscript to meet PLOS ONE’s style requirement.

We have registered a new ORCID ID and validated in Editorial Manager.

We have added Data Availability Statement and submitted the data to NCBI SRA. The RNA-Seq datasets supporting the conclusions of this article are available at NCBI project PRJNA644292 with accession number SRR12153415, SRR12153416, SRR12153417, SRR12153418, SRR12153419, SRR12153420.

We have amended our ethics statement to “The experiments were performed in accordance with the protocol approved by the Institutional Animal Care and Use Committee of Henan Agricultural University and were approved by the Animal Care Committee of the College of Animal Science and Veterinary Medicine, Henan Agricultural University, China (18-0120). In order to complying with animal welfare guidelines and minimize animal suffering. Animals were euthanized with pentobarbitone sodium before tissue sampling.”

We have amended the abstract on the online submission form (via Edit Submission) and the abstract in the manuscript so that they are identical.

Thank you very much.

If there is any question, please let me know.

Looking forward your response!

Best wishes,

Ruili Han
